# Prognostic Analysis of Differentially Expressed DNA Damage Repair Genes in Bladder Cancer

**DOI:** 10.3389/pore.2022.1610267

**Published:** 2022-05-24

**Authors:** Yong Yang, Jieqing Yu, Yuanping Xiong, Jiansheng Xiao, Daofeng Dai, Feng Zhang

**Affiliations:** ^1^ Department of Otolaryngology Head and Neck Surgery, Jiangxi Provincial People’s Hospital Affiliated to Nanchang University, Nanchang, China; ^2^ Department of Otolaryngology Head and Neck Surgery, Jiangxi Provincial People’s Hospital Affiliated to Nanchang Medical College, Nanchang, China; ^3^ Otorhinolaryngology Head and Neck Surgery Institute, Department of Otorhinolaryngology-Head and Neck Surgery, The First Affiliated Hospital of Nanchang University, Nanchang, China; ^4^ Department of General Surgery, The First Affiliated Hospital of Nanchang University, Nanchang, China; ^5^ The National Engineering Research Center for Bioengineering Drugs and the Technologies, The Institute of Translational Medicine, Nanchang University, Nanchang, China

**Keywords:** biomarkers, prognosis, bladder cancer, differentially expressed genes, DNA damage repair genes

## Abstract

Bladder cancer (BCa) is the tenth most common tumor in humans. DNA damage repair genes (DDRGs) play important roles in many malignant tumors; thus, their functions in BCa should also be explored. We performed a comprehensive analysis of the expression profiles of DDRGs in 410 BCa tumors and 19 normal tissues from The Cancer Genome Atlas database. We identified 123 DDRGs differentially expressed between BCa tumors and normal tissues, including 95 upregulated and 28 downregulated genes. We detected 22 DDRGs associated with overall survival (OS) of patients with BCa by performing univariate Cox regression analysis. To explore the interactions between OS-associated DDRGs, we constructed a PPI network, which showed that the top six DDRGs (*CDCA2*, *FOXM1*, *PBK*, *RRM2*, *ORC1*, and *HDAC4*) with the highest scores in the PPI network might play significant roles in OS of BCa. Moreover, to investigate the latent regulatory mechanism of these OS-associated DDRGs, we analyzed the transcription factors (TFs)-DDRGs regulatory network. The core seven TFs (*NCAPG, DNMT1*, *LMNB1*, *BRCA1*, *E2H2*, *CENPA*, and *E2F7*) were shown to be critical regulators of the OS-related DDRGs. The 22 DDRGs were incorporated into a stepwise multivariable Cox analysis. Then, we built the index of risk score based on the expression of 8 DDRGs (*CAD*, *HDAC10*, *JDP2*, *LDLR*, *PDGFRA*, *POLA2*, *SREBF1*, *and STAT1*). The *p*-value < 0.0001 in the Kaplan–Meier survival plot and an area under the ROC curve (AUC) of 0.771 in TCGA-BLCA training dataset suggested the high specificity and sensitivity of the prognostic index. Furthermore, we validated the risk score in the internal TCGA-BLCA and an independent GSE32894 dataset, with AUC of 0.743 and 0.827, respectively. More importantly, the multivariate Cox regression and stratification analysis demonstrated that the predictor was independent of various clinical parameters, including age, tumor stage, grade, and number of positive tumor lymph nodes. In summary, a panel of 8 DNA damage repair genes associated with overall survival in bladder cancer may be a useful prognostic tool.

## Introduction

Cancer is a major global public health problem. Bladder cancer (BCa) is the second most frequent neoplasm of the male urinary tract and is an important cause of death [1]. BCa is now the tenth most common cancer worldwide, with over 573,278 new patients and 212,536 related deaths in 2020 [2]. According to the invasion depth, BCa are divided into non muscle-invasive bladder cancer (NMIBC, Tis, Ta, and T1) and muscle-invasive bladder cancer (MIBC, T2, T3, and T4). BCa is characterized by high morbidity, mortality, and high treatment costs, especially for MIBC [3, 4]. In recent years, although the diagnosis and treatment of BCa have rapidly improved, management of patients remains an important hurdle [5]. BCa is currently classified based on the tumor, lymph node, and metastasis classification system (TNM) for risk stratification and management. However, prediction of the therapeutic effects and outcomes based on the existing treatment strategies remains to be explored [6, 7]. DDRGs are few reported in BCa, and the risk score for molecular subtype of BCa is rarely studied. Thus, it is necessary to find new biomarkers for predicting the outcomes in patients with BCa, which may be useful for improving the management of these patients.

Human cells are challenged with various stresses, including exogenous and endogenous stresses, all of which may result in DNA damage [8, 9]. DNA misrepair can cause mutations that alter the functions of oncogenes and tumor suppressor genes, thus promoting tumorigenesis and tumor development [10]. DDRGs play critical roles in maintaining the genomic stability of human cells. In accordance with biochemical and mechanistic criteria, DDRGs can be grouped into seven capital functional pathways [11].

In this study, we have performed identification and functional analysis of differentially expressed DDRGs, and the selection of those genes that are associated with survival, and the proposal and validation of a prognostic score derived from such genes.

## Materials and Methods

### Clinical Cases and Data Acquisition

First, we downloaded transcriptome RNA sequencing data, including 410 BCa and 19 normal tissue samples and 412 clinical data from The Cancer Genome Atlas (TCGA) database (https://tcga-data.nci.nih.gov/tcga/). The independent validation cohort (referred to as GSE32894) with 308 BCa samples was obtained from Gene Expression Omnibus (GEO) database. We further obtained the list of DDRGs from the GeneCards database (https://pathcards.genecards.org/). BCa was standardized according to the eighth edition of the American Joint Committee on Cancer (AJCC). We subsequently collected individuals or genes based on the following criteria: individuals with survival status and survival time more than 30 days; individuals with a gene expression matrix, and the mean and median of RNA read counts greater than one. At last, there were 392 samples and 18769 RNA from TCGA and 221 patients from GEO for further analysis. The descriptive statistics of clinical features of BCa patients involved in the current study are summarized in Table 1.

**TABLE 1:** The clinicopathological features of BCa patients in TCGA-BLCA dataset and GSE32894 cohort.

Variables	TCGA-BLCA dataset (*n* = 392)	GSE32894 cohort (*n* = 221)	*p*-value
Age			4.00E-02
61–89 years	244 (62.24%)	176 (79.64%)	
34–60 years	97 (24.75%)	45 (20.36%)	
Unknown	51 (13.01%)	—	
Diagnosis subtype			—
Papillary	126 (32.14%)	—	
Non-papillary	261 (66.58%)	—	
Unknown	5 (1.28%)	—	
Gender			8.34E-01
Female	102 (26.02%)	60 (27.15%)	
Male	290 (73.98%)	161 (72.85%)	
Lymph node examined count			—
<=12	78 (19.90%)	—	
>12	178 (45.41%)	—	
Unknown	136 (34.69%)	—	
Lymph nodes			—
Negative	149 (38.01%)	—	
Positive	114 (29.08%)	—	
Unknown	129 (32.91%)	—	
Race			—
White	315 (80.36%)	—	
Black or African American or Asian	61 (15.56%)	—	
Unknown	16 (4.08%)	—	
Tobacco smoking history			—
No	103 (26.28%)	—	
Yes	276 (70.41%)	—	
Unknown	13 (3.31%)	—	
Histologic grade			
Low	18 (4.59%)	130 (58.82%)	2.20E-16
High	371 (94.64%)	91 (41.18%)	
Unknow	3 (0.77%)	—	
Chemotherapy			
No	224 (57.14%)	—	
Yes	139 (35.46%)	—	
not reported	29 (7.40%)	—	
T stage			2.71E-04
I_II	125 (31.89%)	104 (47.06%)	
III_IV	267 (68.11%)	117 (52.94%)	
N stage			—
N0	226	—	
N1-3	130	—	
Unknown	36	—	
M stage			—
M0	187 (47.70%)	—	
M1	10 (2.55%)	—	
Unknown	195 (49.75)	—	

### Identification of Differentially Expressed DNA Damage Repair Genes

The transcriptome data were processed by using R x64 3.6.1 software (https://www.r-project.org/). The R package limma and Wilcox test were applied to obtain the differentially expressed genes. False-discovery rate (FDR) < 0.01 and log_2_|fold change (FC)| > 1 were set as the cutoff value. Differentially expressed DDRGs were extracted from all of the differentially expressed genes, and it was performed as described previously [12].

### Gene Ontology and Kyoto Encyclopedia of Genes and Genomes Pathway Analysis

Gene ontology analysis (GO) was applied to annotate differentially expressed DDRGs. The results of GO analysis were presented in three parts, namely, biological processes (BP), molecular functions (MF), and cellular components (CC). In addition, Kyoto Encyclopedia of Genes and Genomes (KEGG) analysis was used to perform pathway enrichment analysis. Both GO analysis and KEGG analysis were conducted using R x64 3.6.1 software [12].

### Gene Set Enrichment Analysis

As described previously [13], a GSEA version 4.0.1 was first downloaded from the genomic enrichment analysis website (http:/Sofare.Broadstitute.org/GSEA/index.jsp). Then, the differentially expressed genes dataset was imported into the software with reference to the molecular signature database gene set (msigdb.v7.2.symbols gmt). Each analysis was repeated 1,000 times according to the default weighted enrichment statistical method using a genomic error detection rate (FDR) of <0.25 and a pedigree error rate (family‐wise error rate) < 0.05. GSEA analysis included four main statistical data: enrichment score (ES), standardized ES (normalized ES), error detection rate, and *p*-value.

### Construction of Protein-Protein Interaction Network and Hub Genes Selection

In this step, we constructed the PPI network of prognosis-associated differentially expressed DDRGs that had been identified in previous analysis. The Search Tool for the Retrieval of Interacting Genes (STRING) database (version 11.0; https://string-db.org/cgi/input.pl) was used to evaluate the PPI information. Cytoscape software (version 3.8.1) was used to visualize the PPI networks and select hub genes for further discussion [12]. The CBioportal database was used to analyze mutations and copy number variations (CNVs) of differentially expressed DDRGs.

### Univariate and Multivariate Cox Regression Analysis

To identify survival-related genes, we integrated the expression of DDRGs with the OS of BCa patients. The relationships of DDRGs with OS were then analyzed by univariate Cox regression analysis. All samples in TCGA datasets were randomly assigned to the training dataset (60%) and the internal validation dataset (40%). These survival-related DDRGs were integrated into a stepwise multivariate Cox regression to select the optimal model for prognosis [14]. Finally, the prognostic model of BCa was established based on the multivariate co-efficiency multiplied by expression data. The formula was as follows: Risk score = coefficient(a) × gene expression(a) + coefficient(b) × gene expression(b) + ··· + coefficient(n) × gene expression(n). According to the median of risk scores, the patients were divided into high-risk and low-risk groups. The survminer package of R software was used to apply the Kaplan–Meier (KM) curve to investigate the connection between DDRGs and prognosis. The multivariate Cox analysis and stratification analysis were used to explore independent prognostic factors of BCa patients [15].

### TF Analysis and TFs-DDRGs Regulatory Network

The Cistrome Cancer database (http://cistrome.org/CistromeCancer/), a comprehensive database of expression profiles and public ChIP-seq profiles from TCGA, predicts target genes and enhancer profiles of transcription factors (TFs) in TCGA cancer types [16]. Validated TFs were downloaded (318 in total, *p* < 0.05) with statistical relevance to the tumor. These data were combined and the differentially expressed TFs were used to draw a volcano map. Correlation tests between the TFs and prognosis-related DDRGs were performed, and the regulatory network of TFs-DDRGs was presented with Cytoscape [17].

### Statistical Analysis

The data were collected, analyzed, and presented using R software (version 3.6.1) and the corresponding software packages. The performance of the prognostic index was assessed by conducting the receiver operating characteristic (ROC) curve analysis.

## Results

### Identification of Differentially Expressed DDRGs

**FIGURE 1:**
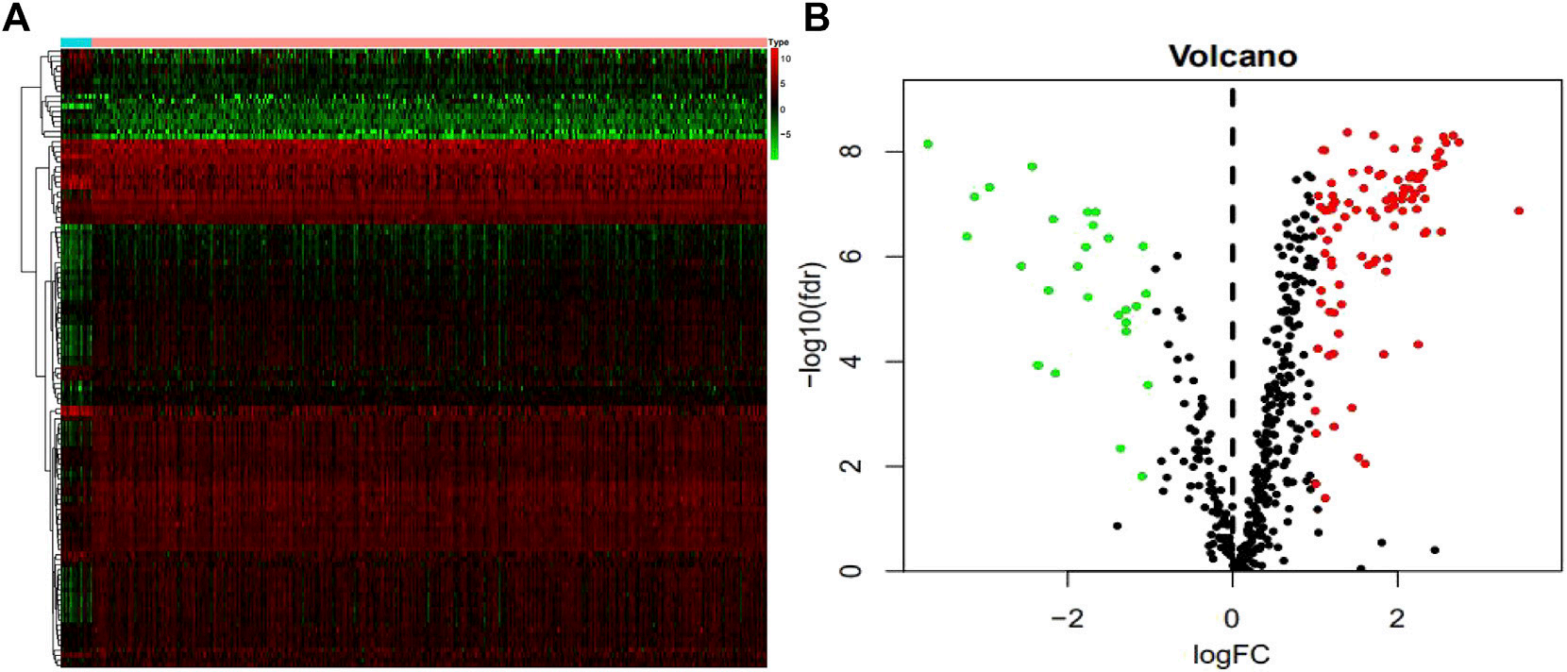
Differentially expressed DNA damage repair genes (DDRGs). Differentially expressed DDRGs are shown in the heat map **(A)** and volcano map **(B)**. The red dot indicates the highly expressed genes, the green dot indicates the low expressed genes, and the black dot indicates the genes without differentially expressed genes.

We studied 410 BCa tissues and 19 normal tissues from the TCGA database. We identified 4,893 differentially expressed genes (DEGs) using the Wilcoxon signed-rank test; 3,468 of them were upregulated and 1,425 were downregulated. There were 123 differentially expressed DDRGs extracted from this set of genes, including 95 upregulated and 28 downregulated genes (Figures 1A,B).

According to gene ontology (GO) analysis in the biological process category, these differentially expressed DDRGs were chiefly enriched in “DNA replication,” “cell division,” and “mitotic nuclear division” (Supplementary Figure S1). GO analysis related to cellular components (CC) showed that they were mainly enriched in “nucleoplasm,” “nucleus,” and “cytoplasm” (Supplementary Figure S1B). GO analysis related to molecular functions (MF) demonstrated that they were involved in “protein binding,” “3′-5′DNA helicase activity,” and “chromatin binding” (Supplementary Figure S1C). KEGG pathway analysis revealed that “cell cycle,” “homologous recombination,” and “p53 signaling pathway” were the most enriched pathways (Supplementary Figure S1D). GSEA of the DEGs revealed that these genes were enriched in “SHEDDEN_LUNG_CANCER_POOR_SURVIVAL_A6”, “GOBERT_OLIGODENDROCYTE_DIFFERENTIATION_UP”, and “MARSON_BOUND_BY_E2F4_UNSTIMULATED” (Supplementary Figure S2).

### Identification of Survival-Associated DDRGs

To explore the prognostic potential of the differentially expressed DDRGs in BCa patients, we directed our efforts toward uncovering molecular biomarkers. First, we downloaded the clinical information of patients with BCa. By univariate Cox regression analysis, the top 22 differentially expressed DDRGs that were considered relevant with OS (Table 2). Most of these genes had positive hazard ratios, which indicated a higher risk for BCa patients.

**TABLE 2:** The prognostic value of differentially expressed DDRGs by univariate Cox regression analysis.

Gene	HR	95% CI	*p*-value
ATXN1	1.183	1.032–1.356	0.016
CAD	1.055	1.026–1.085	0.000
CDCA2	1.075	1.009–1.145	0.024
CDK5R1	1.064	1.007–1.124	0.026
FOXM1	1.020	1.004–1.036	0.011
HDAC10	0.847	0.764–0.939	0.002
HDAC4	1.366	1.103–1.691	0.004
ISG15	0.999	0.998–1.000	0.047
JDP2	1.059	1.013–1.106	0.012
LATS2	1.092	1.019–1.170	0.013
LDLR	1.021	1.007–1.035	0.003
MT1A	1.015	1.007–1.023	0.000
NEIL3	1.141	1.016–1.282	0.026
ORC1	1.071	1.004–1.141	0.036
PBK	1.022	1.002–1.041	0.027
PDGFRA	1.045	1.018–1.073	0.001
POLA2	1.066	1.005–1.130	0.033
RRM2	1.006	1.000–1.012	0.037
SREBF1	1.007	1.000–1.013	0.046
STAT1	0.996	0.992–0.999	0.015
TACC1	1.013	1.001–1.024	0.026
THBS1	1.004	1.001–1.007	0.009

**FIGURE 2:**
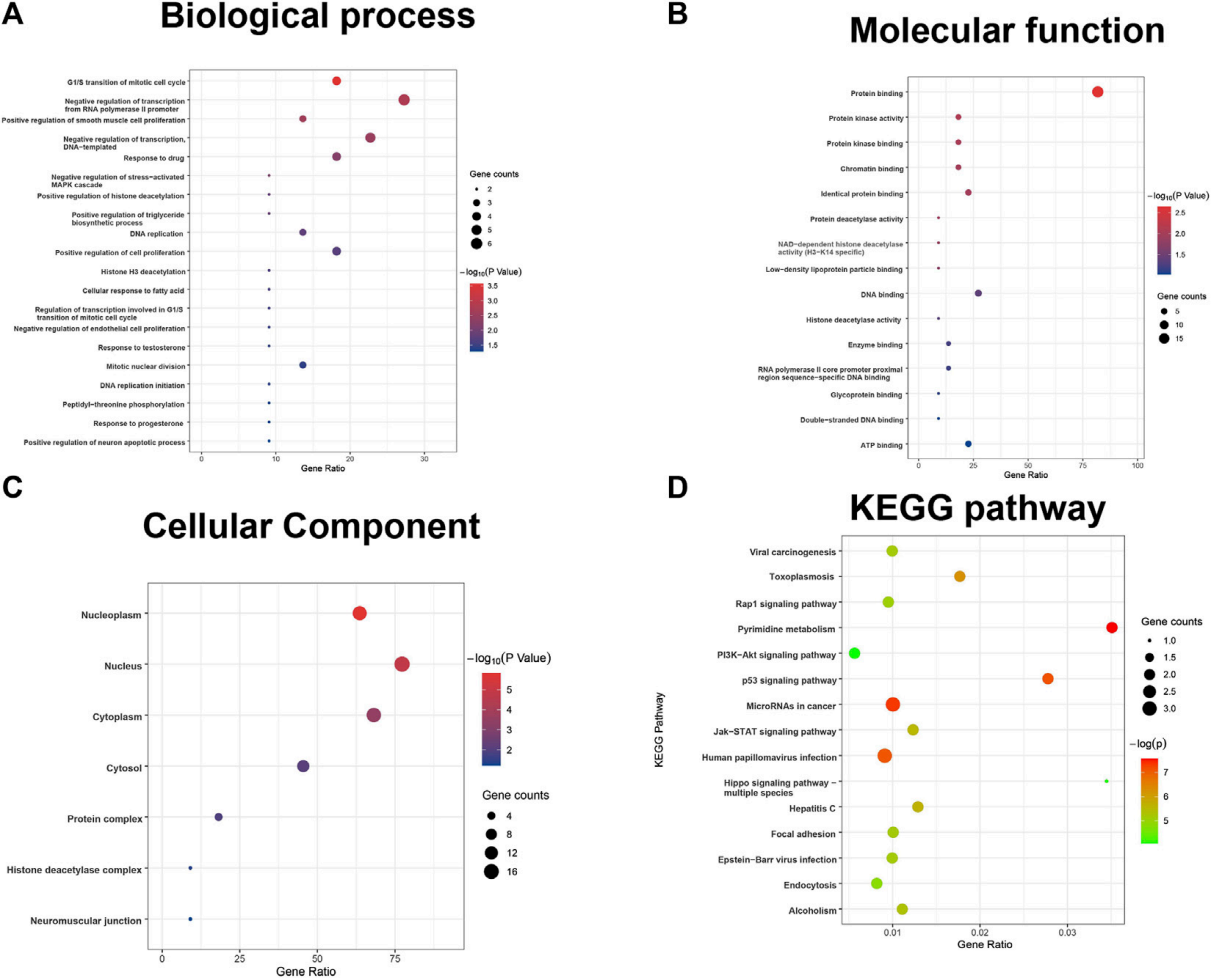
GO terms and pathways analysis of the differentially expressed overall survival (OS)-related DDRGs. **(A)** Gene ontology analysis: biological processes. **(B)** Gene ontology analysis: molecular functions. **(C)** Gene ontology analysis: cellular components. **(D)** The significant enriched KEGG pathways.

Furthermore, the results of the enrichment analysis of the differentially expressed DDRGs showed that OS-related DDRGs were significantly enriched in similar GO terms and KEGG pathways, such as “negative regulation of transcription from RNA polymerase II promoter,” “G1/S transition of mitotic cell cycle,” and “positive regulation of smooth muscle cell proliferation” (Figure 2A). Also, “protein binding,” “protein kinase activity,” and “protein kinase binding” (Figure 2B) were the most significant MF. Furthermore, “nucleoplasm,” “nucleus,” and “cytoplasm” (Figure 2C) were the most significant CC. In addition, “Human papillomavirus infection,” “Pyrimidine metabolism,” and “MicroRNAs in cancer” were the most significant pathways (Figure 2D).

### Construction of Protein–Protein Interaction Network Based on Prognostic DDRGs

PPI network analysis of OS-associated differentially expressed DDRGs was performed using STRING (version 11.0) and visualized using Cytoscape (version 3.8.1). It showed that *PBK, CDCA2, FOXM1, RRM2*, *ORC1*, and *HDAC4* were the hub genes (Supplementary Figure S3). According to mutation and CNV analysis for BCa performed using the cBioportal database, the three most common types of OS-related DDRGs included amplification, missense mutations, and deep deletions (Supplementary Figure S4). We also performed the regression analysis of risk score on tumor mutational burden with the *p*-value of 0.96. The scatter plot was shown in Supplementary Figure S7.

### Construction of Transcription Factors Regulatory Network

**FIGURE 3:**
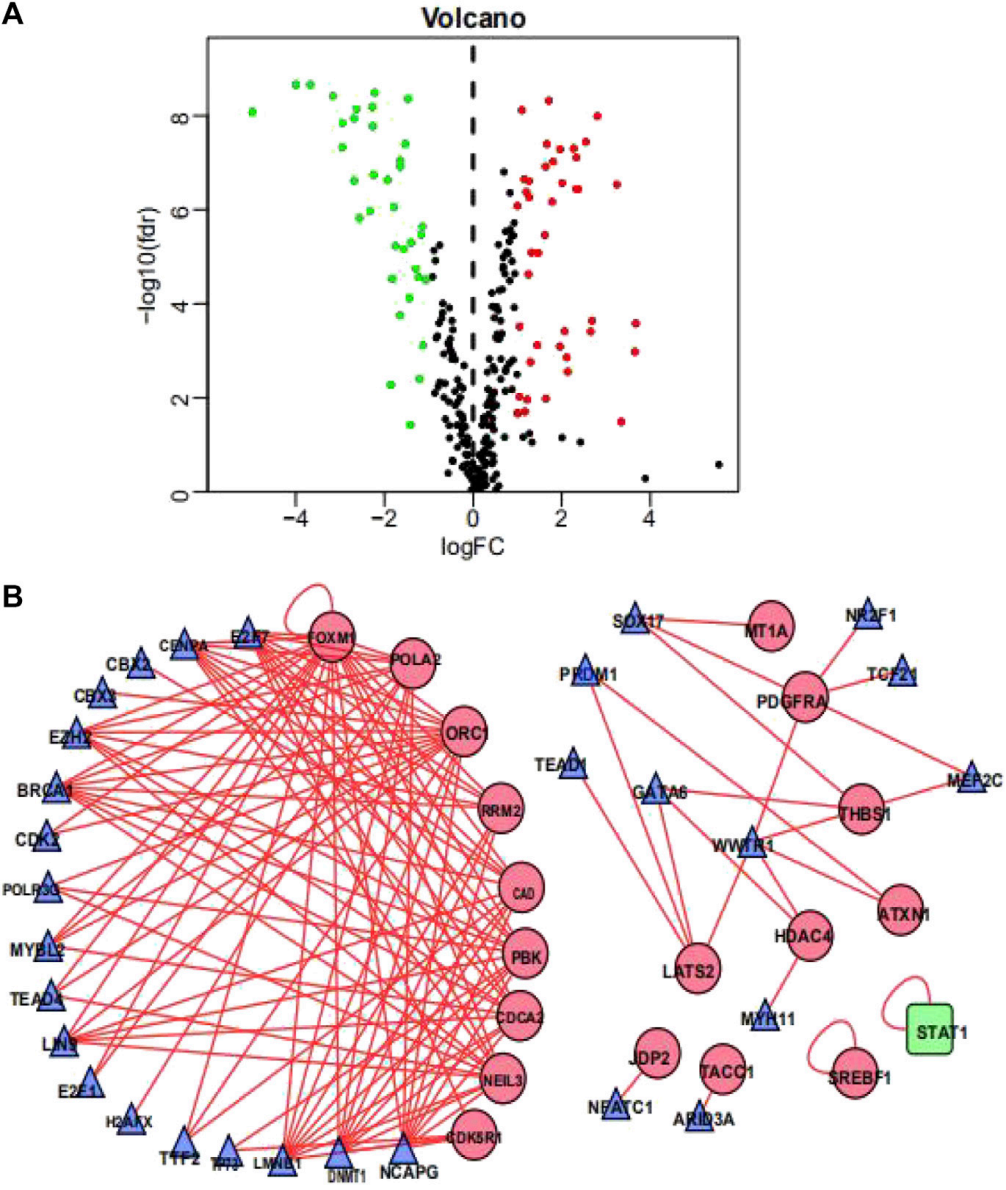
Transcription factors-mediated regulatory network. **(A)** Volcano plot of differentially expressed Transcription Factors (TFs)between BCa and non-tumors tissues. **(B)** The transcription regulatory network according to the clinically relevant DDRGs and differentially expressed TFs. The circle in a node reflects clinically relevant DDRGs and triangle represented as differentially expressed TFs. The shades of color reflect the correlation.

To investigate the possible molecular mechanisms regulating the OS-related DDRGs, we analyzed the TF-DDRG interaction. First, we observed the expression profiles of 318 TFs; there were 77 TFs that were differentially expressed between BCa tissues and non-tumor tissues. Among them, 41 were upregulated and 33 were downregulated (Figure 3A). We then constructed a regulatory network based on these 77 TFs and 22 OS-related DDRGs. We set cutoff thresholds as correlation scores >0.4 and *p*-values < 0.001. The TF-based regulatory network is shown in Figure 3B. We identified the key regulated factors of OS-related DDRGs using the TRRUST database. Seven key transcription factors (*NCAPG*, *DNMT1*, *LMNB1*, *BRCA1*, *E2H2*, *CENPA*, and *E2F7*) were found to be associated with the regulation of these DDRGs.

### Development and Validation of the DNA Damage Repair Prognostic Index

**FIGURE 4:**
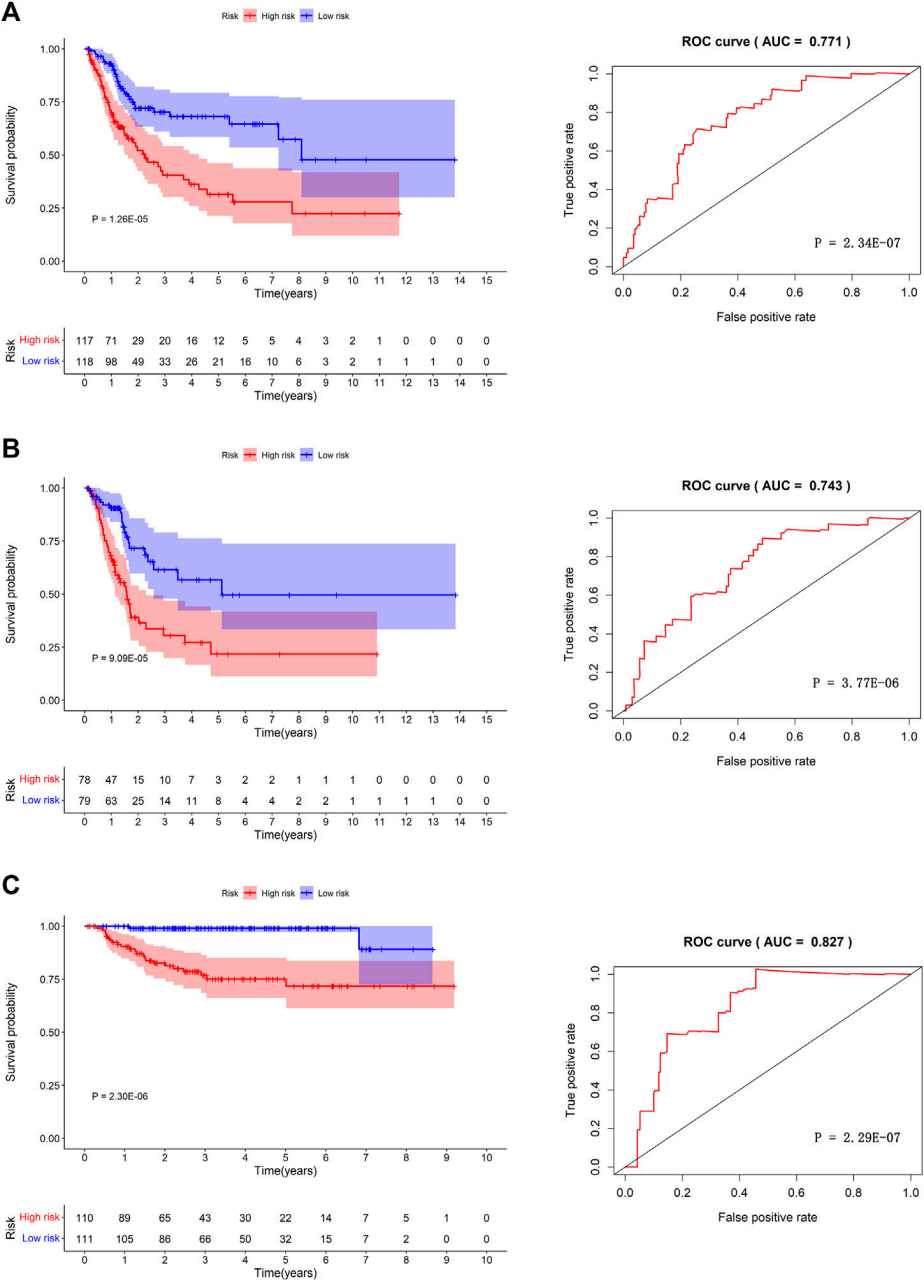
The prognosis model in TCGA-BLCA training dataset, TCGA-BLCA internal validation dataset and GSE32894 independent validation cohort. Kaplan-Meier survival curves between high-risk group and low-risk group (left) and the receiver operating characteristic (ROC) curve of the risk scores (right) in TCGA-BLCA training dataset **(A)**, TCGA-BLCA internal validation dataset **(B)**, and GSE32894 validation cohort **(C)**.

We separated the TCGA-BLCA cohort into training dataset (60%) and internal validation dataset (40%) by random selection. The following optimal model was chosen by a stepwise multivariate Cox analysis: [Expression level of *CAD* * (0.042)] + [Expression level of *HDAC*10*(−0.146)] + [Expression level of *JDP2* * (0.069)] + [Expression level of *LDLR* * (0.022) + [Expression level of *PDGFRA* * (0.070)] + [Expression level of *POLA2* * (0.108)] + [Expression level of *SREBF1* * (0.008)] + [Expression level of *STAT1* *(−0.009)] (Table 3). We built a prognostic index-risk score to divide patients with BCa into two groups, the high-risk group and the low-risk group, and then we made a risk curve. The KM analysis showed that there was a statistically significant difference in OS between the two groups with *p*-value < 0.0001 (Figure 4A). A higher risk score was associated with a shorter survival time. The area under the ROC curve (AUC) was 0.771, *p* = 2.34E-07 (Figure 4A), which indicated a high forecast ability of the DDRG-based risk score in survival surveillance. The prognostic index was confirmed in TCGA-BLCA internal validation dataset, with a similar KM plot (*p*-value <0.0001) and ROC curve (AUC = 0.743, *p* = 3.77E-06) (Figure 4B). The survival status and risk scores of each BCa patient are listed in Supplementary Figure S5A for the TCGA-BLCA training dataset, Supplementary Figure S5B for the internal validation dataset, and Supplementary Figure S5C for the GSE32894 cohort.

**TABLE 3:** Multivariate cox analysis to develop a prognostic index based on these differentially expressed DNA damage repair genes.

Gene	coef	HR	95% CI	*p*-value
CAD	0.042	1.043	0.989–1.100	0.117
HDAC10	−0.146	0.864	0.759–0.984	0.028
JDP2	0.069	1.071	1.014–1.131	0.014
LDLR	0.022	1.022	1.000–1.045	0.049
PDGFRA	0.070	1.073	1.003–1.147	0.039
POLA2	0.108	1.114	1.015–1.221	0.022
SREBF1	0.008	1.008	1.000–1.017	0.057
STAT1	−0.009	0.991	0.985–0.996	0.001

Furthermore, we validated the performance of the prognostic model in an independent dataset, GSE32894, through the KM survival plot and ROC curve analysis. A significant *p*-value < 0.0001 in the KM survival analysis and an area under the ROC curve of 0.827 verified the efficiency of our prognostic model, *p* = 2.29E-07 (Figure 4C). To compare the prediction ability of risk score with clinical features, we conducted ROC analyses for each clinical feature (Supplementary Figure S6A) and combined clinical features and risk scores (Supplementary Figure S6B). The results showed that the AUC of the prognostic index was larger than those based on age, count of examined lymph nodes, positive lymph nodes, and tumor stage (Supplementary Figure S6A). When combining clinical features and risk scores, we found that our prognostic index could improve the clinical prognostication (Supplementary Figure S6B).

### Correlation Between the Prognostic Index and Other Clinicopathologic Characteristics

**FIGURE 5:**
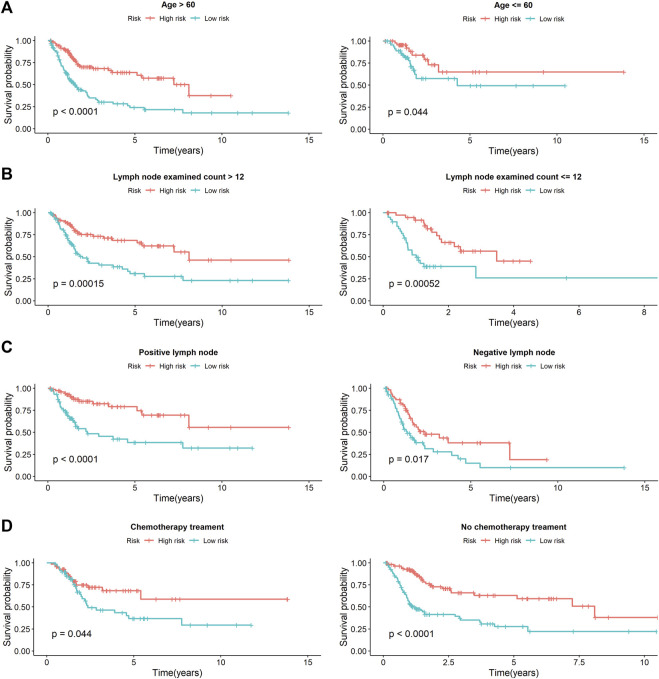
The stratification analysis of prognostic signature in BCa patients with different clinical parameters. **(A)** The prognostic utility of the signature in BCa patients with different age groups. **(B)** The prognostic utility of the signature in BCa patients with number of lymph nodes. **(C)** The prognostic utility of the signature in BCa patients with number of positive lymph nodes. **(D)** The prognostic utility of the signature in BCa patients with tumor mutational burden.

Univariate Cox analysis revealed that age, number of lymph nodes, number of positive lymph nodes, stage, and risk scores were related to OS. After adjusting for age, gender, race, tobacco smoking history, number of lymph nodes, number of positive lymph nodes, and clinical stage by multivariate Cox analysis, the risk score was still associated with OS, with a *p*-value <0.001 (Table 4). However, we noticed that age, number of lymph nodes, and number of positive lymph nodes were also related to OS (*p* < 0.05). In addition, chemotherapy treatment is an important factor to OS. To further confirm the independence of the risk scores, we performed a stratification analysis. All of the patients were separated into two subgroups as shown in Table 1. We found that the risk scores still correlated with OS (*p* < 0.05, Figure 5).

**TABLE 4:** The Cox regression analysis of clinical characteristics and the prognostic signature in TCGA-BLCA cohort.

Variables	Untivariate analysis			Multivariate analysis		
	HR	95% CI	*p*-value	HR	95% CI	*p*-value
risk-group (high vs. low)	2.67	1.90–3.74	1.17E-08	2.39	1.25–4.57	8.24E-03
age (>60 vs. <=60)	1.98	1.26–3.11	2.89E-03	2.11	0.91–4.92	8.30E-02
diagnosis subtype (non-papillary vs. papillary)	1.85	1.22–2.79	3.54E-03	1.05	0.45–2.46	9.15E-01
gender (male vs. female)	0.85	0.6–1.21	3.79E-01	—	—	—
race (not white vs. white)	0.86	0.52–1.43	5.58E-01	—	—	—
tobacco smoking history (yes vs. no)	1.43	0.97–2.1	7.19E-02	—	—	—
lymph node examined count (>12 vs. <=12)	0.63	0.42–0.95	2.61E-02	0.37	0.18–0.75	6.08E-03
lymph nodes (positive vs. negative)	2.16	1.48–3.15	6.49E-05	1.73	0.74–4.05	2.05E-01
stage T (III_IV vs. I_II)	2.67	1.74–4.1	7.53E-06	2.06	0.69–6.16	1.96E-01
stage N (N1-3 vs. N0)	2.42	1.72–3.4	3.39119E-07	1.40	0.57–3.42	4.59E-01
stage M (M1 vs. M0)	2.67	1.15–6.22	2.27E-02	0.64	0.07–6.1	6.96E-01

We additionally accessed the correlation between DDRG index and subtypes in TCGA and GEO database (*p* < 0.001, Supplementary Figure S8). Except SREBF1, other DDRGs were correlated with diagnosis subtypes between Non-Papillary and Papillary (Table 5). Except JDP2, the expression of each gene was related to the molecular subtypes (Table 6).

**TABLE 5:** Difference analysis of gene expression between non papillary and papillary in the TCGA database.

Variables	Total (*n* = 387)	Group		Statistics	*p*
		Non-Papillary (*n* = 261)	Papillary (*n* = 126)		
CAD, M(Q_1_,Q_3_)	8.74 (6.28, 12.31)	9.38 (6.93, 12.86)	7.31 (5.63, 11.35)	Z = -3.408	**<0.001**
HDAC10, M(Q_1_,Q_3_)	2.54 (1.81, 3.96)	2.29 (1.72, 3.57)	3.28 (1.99, 4.87)	Z = 4.043	**<0.001**
JDP2, M(Q_1_,Q_3_)	3.09 (1.77, 5.03)	3.45 (2.10, 5.39)	2.30 (1.18, 4.01)	Z = -4.920	**<0.001**
LDLR, M(Q_1_,Q_3_)	7.04 (3.32, 12.75)	7.43 (3.59, 13.33)	5.80 (2.83, 11.72)	Z = -2.211	**0.027**
PDGFRA, M(Q_1_,Q_3_)	1.66 (0.75, 3.45)	1.78 (0.90, 3.71)	1.30 (0.51, 3.16)	Z = -2.413	**0.016**
POLA2, M(Q_1_,Q_3_)	4.95 (3.65, 6.67)	5.10 (3.90, 6.68)	4.59 (3.23, 6.53)	Z = -2.387	**0.017**
SREBF1, M(Q_1_,Q_3_)	18.51 (12.17, 32.91)	17.43 (11.57, 32.26)	20.60 (12.98, 35.93)	Z = 1.442	0.149
STAT1, M(Q_1_,Q_3_)	33.14 (17.59, 70.31)	39.10 (19.95, 80.48)	22.07 (13.26, 49.97)	Z = -4.707	**<0.001**
riskScore, M(Q_1_,Q_3_)	0.95 (0.59, 1.51)	1.02 (0.66, 1.63)	0.74 (0.49, 1.31)	Z = -3.604	**<0.001**
risk, n (%)				χ^2^ = 11.806	**<0.001**
High	193 (49.87)	146 (55.94)	47 (37.30)		
Low	194 (50.13)	115 (44.06)	79 (62.70)		

**TABLE 6:** Difference analysis of gene expression among the molecular subtypes in the GEO database.

Variables	Total (*n* = 221)	Group					Statistics	*p*
		SCC-like (*n* = 11)	genomically unstable (*n* = 55)	infiltrated (*n* = 31)	Urobasal A (*n* = 110)	Urobasal B (*n* = 14)		
CAD, M(Q_1_,Q_3_)	−0.04 (−0.27, 0.23)	0.40 (−0.24, 0.71)	0.21 (−0.12, 0.44)	−0.02 (−0.27, 0.23)	−0.13 (−0.32, 0.09)	−0.14 (−0.30, 0.04)	χ^2^ = 26.152	**<0.001**
HDAC10, M(Q_1_,Q_3_)	0.00 (−0.15, 0.16)	−0.09 (−0.21, −0.04)	−0.11 (−0.21, 0.05)	−0.05 (−0.20, 0.06)	0.10 (−0.04, 0.28)	−0.06 (−0.19, 0.08)	χ^2^ = 35.819	**<0.001**
JDP2, M(Q_1_,Q_3_)	−0.05 (−0.19, 0.12)	0.01 (−0.12, 0.13)	−0.05 (−0.18, 0.12)	−0.03 (−0.16, 0.12)	−0.06 (−0.20, 0.14)	−0.01 (−0.07, 0.09)	χ^2^ = 1.636	0.802
LDLR, M(Q_1_,Q_3_)	0.05 (−1.30, 0.76)	0.58 (0.26, 1.33)	−0.02 (−0.93, 0.76)	0.44 (−0.58, 1.13)	−0.47 (−1.78, 0.37)	0.81 (0.39, 1.45)	χ^2^ = 24.410	**<0.001**
PDGFRA, M(Q_1_,Q_3_)	−0.24 (−0.83, 0.52)	0.22 (0.11, 0.80)	−0.30 (−0.96, 0.31)	1.44 (0.59, 2.12)	−0.60 (−0.99, 0.14)	0.02 (−0.59, 0.56)	χ^2^ = 58.297	**<0.001**
POLA2, M(Q_1_,Q_3_)	−0.17 (−0.45, 0.37)	0.46 (0.04, 0.80)	0.52 (0.16, 0.73)	−0.22 (−0.39, 0.14)	−0.40 (−0.57, −0.17)	0.09 (−0.17, 0.53)	χ^2^ = 93.192	**<0.001**
SREBF1, M(Q_1_,Q_3_)	0.05 (−0.48, 0.58)	0.09 (−0.33, 0.66)	0.14 (−0.32, 0.90)	−0.60 (−0.81, −0.11)	0.05 (−0.32, 0.56)	0.55 (−0.19, 0.66)	χ^2^ = 19.973	**<0.001**
STAT1, M(Q_1_,Q_3_)	−0.21 (−0.87, 0.66)	1.31 (0.66, 2.11)	−0.19 (−0.88, 0.76)	0.36 (−0.15, 1.44)	−0.59 (−1.04, −0.07)	0.85 (0.28, 1.43)	χ^2^ = 57.366	**<0.001**
risk_score, M(Q_1_,Q_3_)	−0.02 (−0.11, 0.05)	0.13 (0.02, 0.16)	0.04 (−0.03,0.08)	0.06 (0.03, 0.14)	−0.10 (−0.15, −0.03)	0.03 (−0.02, 0.08)	χ^2^ = 109.387	**<0.001**
risk_group, n (%)							χ^2^ = 80.861	**<0.001**
High	110 (49.77)	10 (90.91)	37 (67.27)	30 (96.77)	23 (20.91)	10 (71.43)		
Low	111 (50.23)	1 (9.09)	18 (32.73)	1 (3.23)	87 (79.09)	4 (28.57)		

## Discussion

Deficiency in DDR pathways is an early and critical step in tumorigenesis and plays an important role in tumor progression and response to platinum-based systemic therapy [18, 19]. With the development of genome sequencing, some novel molecular biomarkers have been screened out to predict the prognosis and offer personalized treatment guidance for reference.

In the present study, we performed a comprehensive integrated analysis of DDRGs and interpreted their clinical traits in patients with BCa. With the improvement of medical treatment and genome-sequencing techniques, a better understanding of human tumor occurrence and progression has been achieved [20]. Instead of conventional clinical-pathological subtypes, molecular subtypes of cancers have gained increasing attention in the prognosis of patients [21]. Molecular subtypes of BCa might have an important role in predicting prognosis and guiding clinical therapy [22–24].

Up to now, there have been a number of predicting models or indexes for BCa patients. Alessandra Allione et al. [25] suggested that DNA damage levels measured in peripheral blood mononuclear cells of patients with BCa may potentially represent a prognostic marker associated with poor survival. Duan et al. [26] developed a panel for diagnosis based on three lncRNAs in serum, which was confirmed to perform better than urine cytology. Fang et al. [27] found that miR-205 may be a promising biomarker for the detection and prognosis evaluation of BCa. MierXiati A et al. [28] prospectively built and validated a 12-gene signature for the survival of non-muscle invasive bladder cancer (NMIBC) and found that the prognostic power of this score was superior to that of clinical data. Ingelmo-Torres et al. [29] constructed a predicting model based on two urinary cell microRNAs, miR-140-5p and miR-92a-3p. Chen et al. [30] identified a four-gene signature that was useful in overall survival prediction in patients with urinary bladder cancer. The selected four genes might become potential therapeutic targets and diagnostic markers for urinary bladder cancer.

However, few studies have focused on the DNA damage repair of BCa, and there have been no robust prognostic models based on DDR. In this study, we established a model of DDRGs to predict the prognosis of BCa patients and explored the underlying mechanism.

In our study, univariate Cox regression analysis showed that 22 DDRGs were significantly associated with OS, indicating that those genes could be very important prognostic factors for patients with BCa. The functions of OS-related DDRGs are listed in Supplementary Table S1.

To explore the possible molecular mechanisms, we used a TF-mediated network to identify the TFs that might mediate these DDRGs. The TF-DDRG regulatory network might be helpful to guide future mechanistic studies. We found seven core TFs, namely, *NCAPG, DNMT1, LMNB1, BRCA1, E2H2, CENPA,* and *E2F7,* to be critical regulators of the OS-related DDRGs. The functions of the seven TFs are listed in Supplementary Table S2.

In this study, through multivariate Cox regression analysis, we first identified a set of 8 DDRGs (*CAD*, *HDAC10*, *JDP2*, *LDLR*, *PDGFRA*, *POLA2*, *SREBF1*, and *STAT1*) that were significantly associated with OS of BCa patients. We also developed a new prognostic index. The overall survival of patients with low risk was significantly higher compared with that in patients with high risk. The AUC of ROC was 0.743 in TCGA-BLCA internal validation dataset, suggesting the potential of this prognostic index. Furthermore, we performed an independent validation to validate our prognostic signature in the GSE32894 cohort. The results demonstrated the robustness of our model, with *p*-value <0.0001 in the survival plot and an area under the ROC curve of 0.827. Both TCGA validation and GEO validation suggested the predictive power of this index. Due to the characteristical difference between TCGA dataset only including MIBC patients and GEO cohort including NMIBC and MIBC samples, we removed the NMIBC patients and re-validated the prognostic signature in GSE32894 cohort. The KM plot represented that the high and low DDRG index group would not significantly estimate the OS with *p*-value of 0.15 (data not shown), which may be caused by the heterogeneity and complexity of the transcriptome profile of MIBC patients in the two datasets.

Multivariate Cox analysis and stratification analysis demonstrated that the risk score was an independent predictor for bladder cancer by controlling the potential confound clinical factors. Notably, DDRG index was proved to be associated with poor survival OS both in the chemotherapy and non-chemotherapy subgroups. After clinical correction, we constructed prognostic markers, which may be useful to be independent predictors; they also showed high clinical practicability to predict the development of BCa. The confirmation of a BCa prognosis index based on DDRGs provides evidence for appropriate clinical therapy, which is not only useful for assessment of patient terms, but it is also helpful for further understanding the functions of DDRGs. Nevertheless, this requires further research.

In our study, according to diagnosis subtype, we have divided BCa into non-Papillary and Papillary groups. Unfortunately, the diagnosed subtype was not strongly related to BCa’ survival time. Compared to previously proposed molecular subtyping, DDRG risk score that we have proposed may be more convincing. Because the AUC of DDRG risk score is 0.827, which is higher than previous study (the AUC is 0.761) [30]. Meanwhile, this study has few limitations. First, we obtained all of the data of this study from a public database, and the number of samples in the public database was limited. Second, transcriptomic analysis can only reflect certain aspects of the DNA damage status, not global alterations. Third, this was a retrospective study, and a multicenter and prospective study is needed to validate our results by *in vitro* and *in vivo* experiments.

## Conclusion

In this study, we have performed identification and functional analysis of differentially expressed DDRGs, and the selection of those genes that are associated with survival, and the proposal and validation of a prognostic score derived from such genes. In summary, a panel of 8 DNA damage repair genes associated with overall survival in bladder cancer may be a useful prognostic tool.

## Data Availability

The datasets presented in this study can be found in online repositories. The names of the repository/repositories and accession number(s) can be found in the article/Supplementary Material.

## Supplementary Material

Supplementary Figure S1 Gene function enrichment and pathway analysis of the differentially expressed DNA damage repair genes (DDRGs). **(A)** Gene ontology analysis: biological processes. **(B)** Gene ontology analysis: molecular functions. **(C)** Gene ontology analysis: cellular components. **(D)** The significant enriched KEGG pathways.

Supplementary Figure S2 Gene set enrichment analysis (GSEA) of the differentially expressed OS-related DNA damage repair genes (DDRGs). Only listed the top 8 significantly enriched pathways ES, enrichment score; FDR, false discovery rate; GSEA, gene set enrichment analysis.

Supplementary Figure S3 Protein interaction network of overall survival (OS)-related DNA damage repair genes. **(A)** Protein–protein interaction network constructed through STRING database based on prognostic DNA damage repair genes (DDRGs). **(B)** Protein–protein interaction network of prognostic DNA damage repair genes built based on connectivity using Cytoscape.

Supplementary Figure S4 Mutation landscape of OS-related differentially expressed DDRGs.

Supplementary Figure S5 The distribution of risk scores based on DDRGs in TCGA-BLCA dataset and GSE32894 cohort. **(A)** The distribution of patients’ risk scores (top) and survival time (bottom) in high risk and low risk groups based on TCGA-BLCA training dataset. **(B)** The distribution of patients’ risk scores (top) and survival time (bottom) in high risk and low risk groups based on TCGA-BLCA internal validation dataset. **(C)** The distribution of patients’ risk scores (top) and survival time (bottom) in high risk and low risk groups based on GSE32894 dataset.

Supplementary Figure S6 The comparison of ROC curve analysis for different clinical features and prognotic index.

Supplementary Figure S7 The linear regression of risk score on tumor mutational burden.

Supplementary Figure S8 The boxplot of risk scores in different subtypes. **(A)** The boxplot of risk scores in diagnosis subtypes based on the TCGA-BLCA dataset. **(B)** The boxplot of risk scores in the molecular subtypes based on the GSE32894 cohort.
