# Supplementary figures and images for: Prognostic Analysis of Differentially Expressed DNA Damage Repair Genes in Bladder Cancer
Source: Pathol Oncol Res. 2022 May 24;28:1610267. doi: 10.3389/pore.2022.1610267 (PMC9172279; doi:10.3389/pore.2022.1610267)

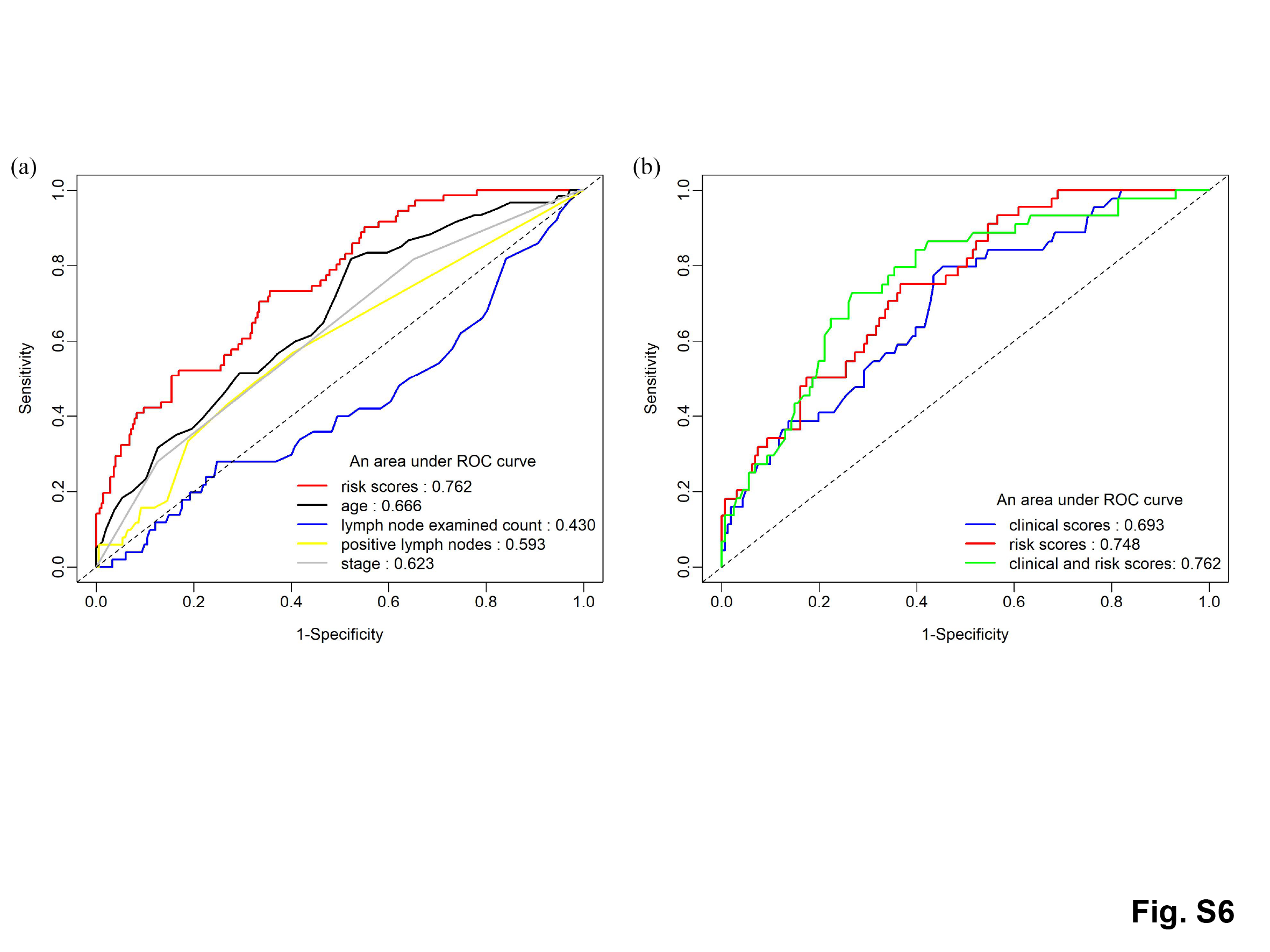

Supplement: Supplementary file 1 [file Image6.TIF]

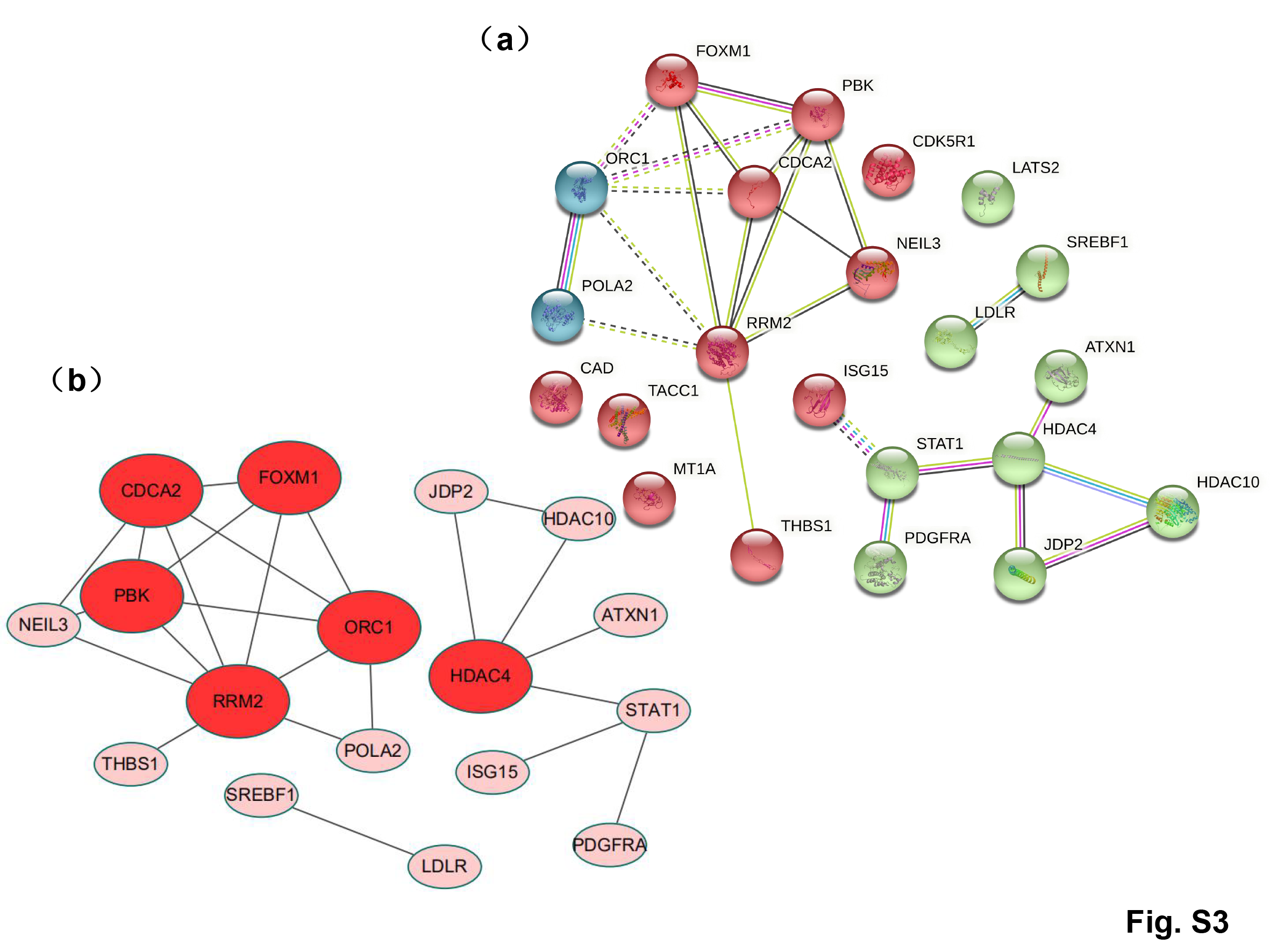

Supplement: Supplementary file 2 [file Image3.TIF]

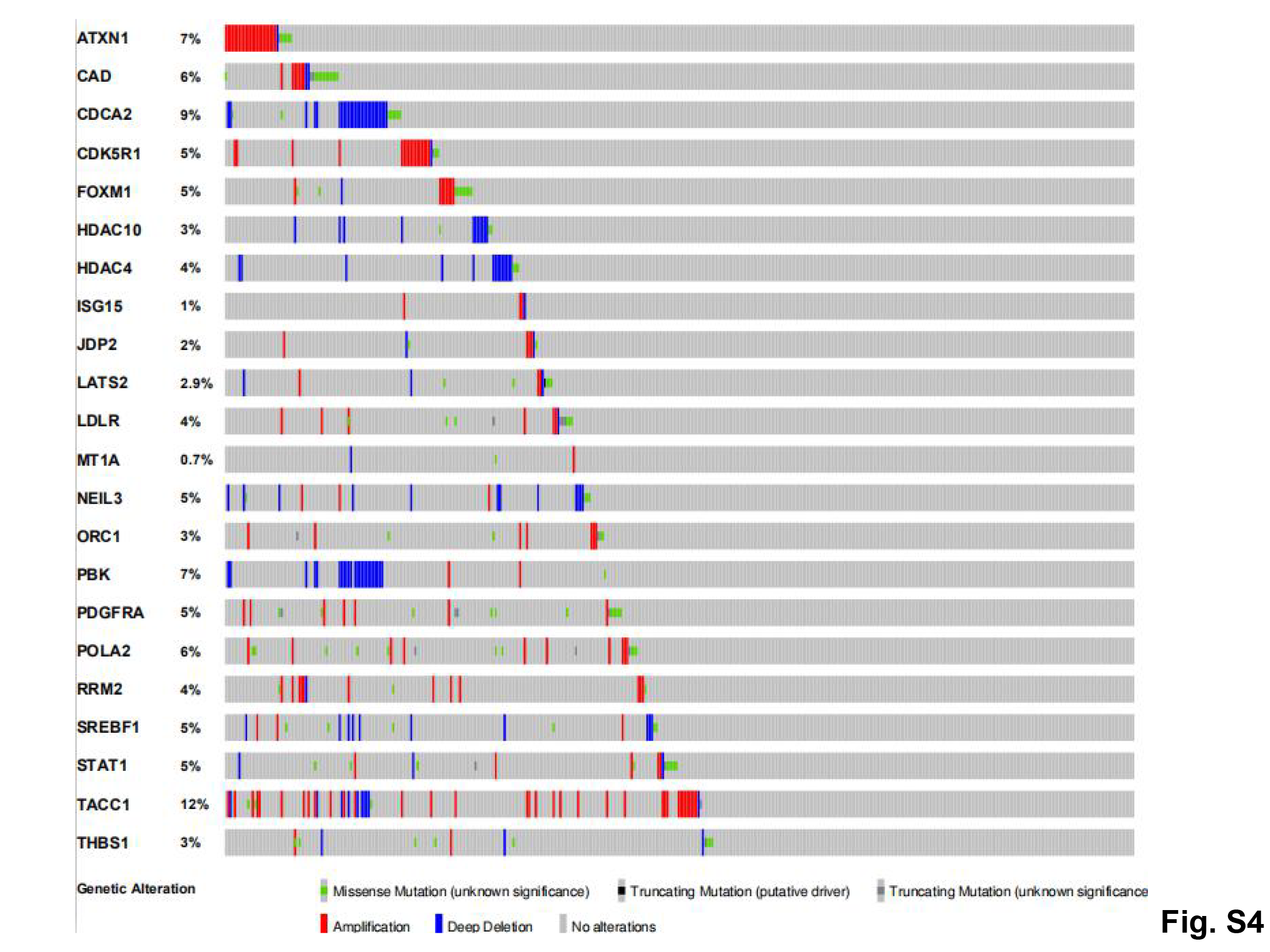

Supplement: Supplementary file 3 [file Image4.TIF]

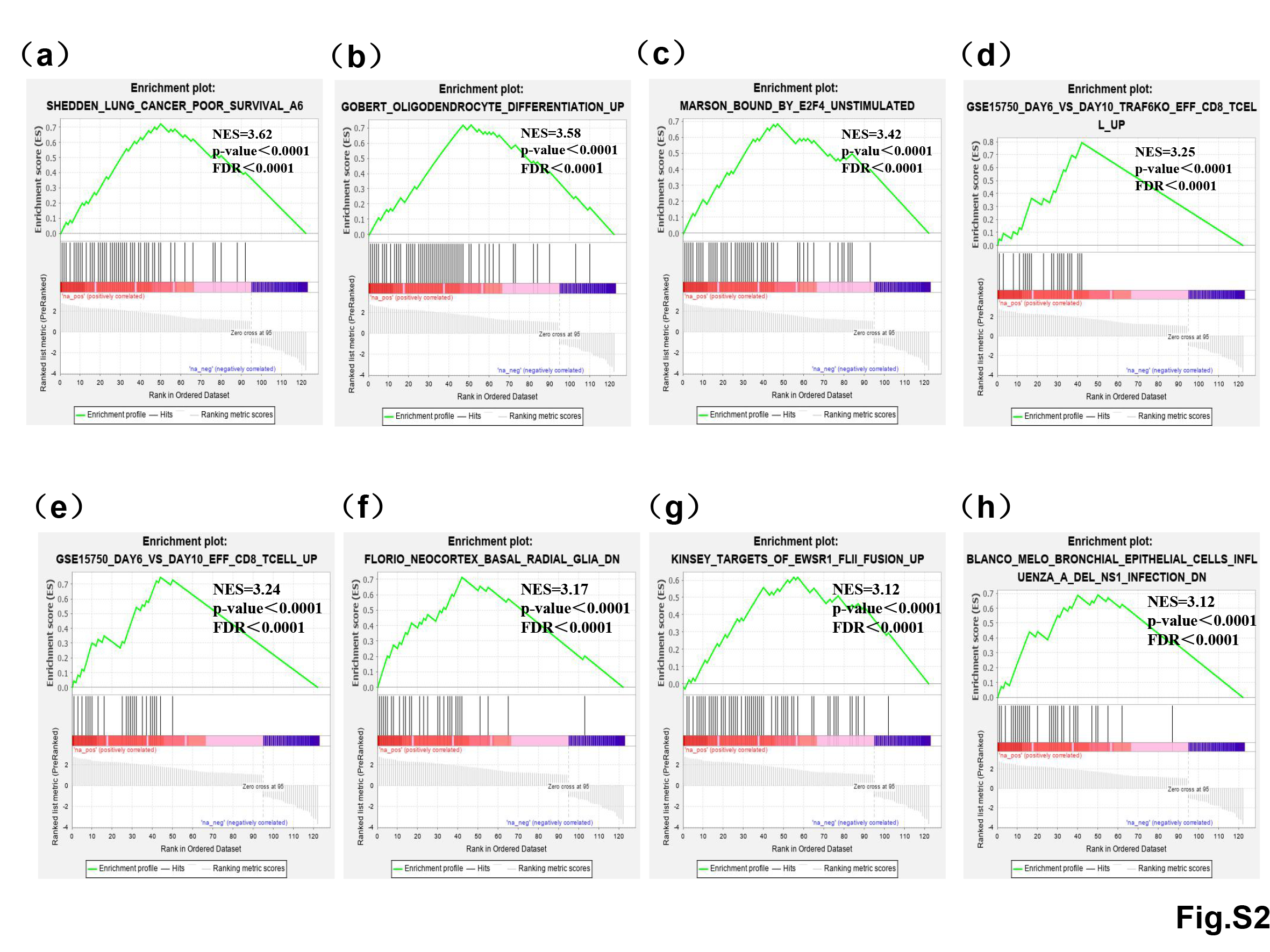

Supplement: Supplementary file 4 [file Image2.TIF]

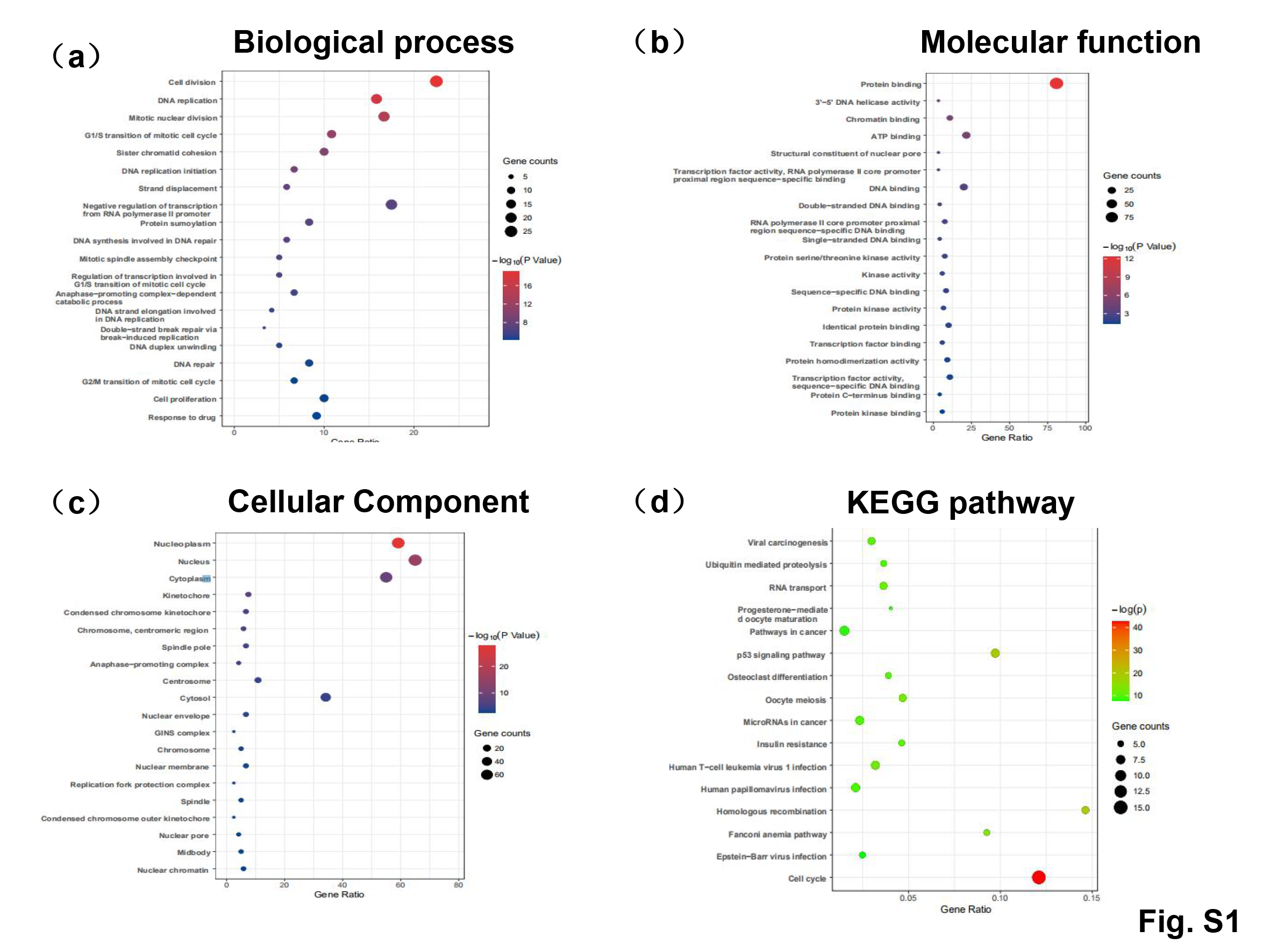

Supplement: Supplementary file 5 [file Image1.TIF]

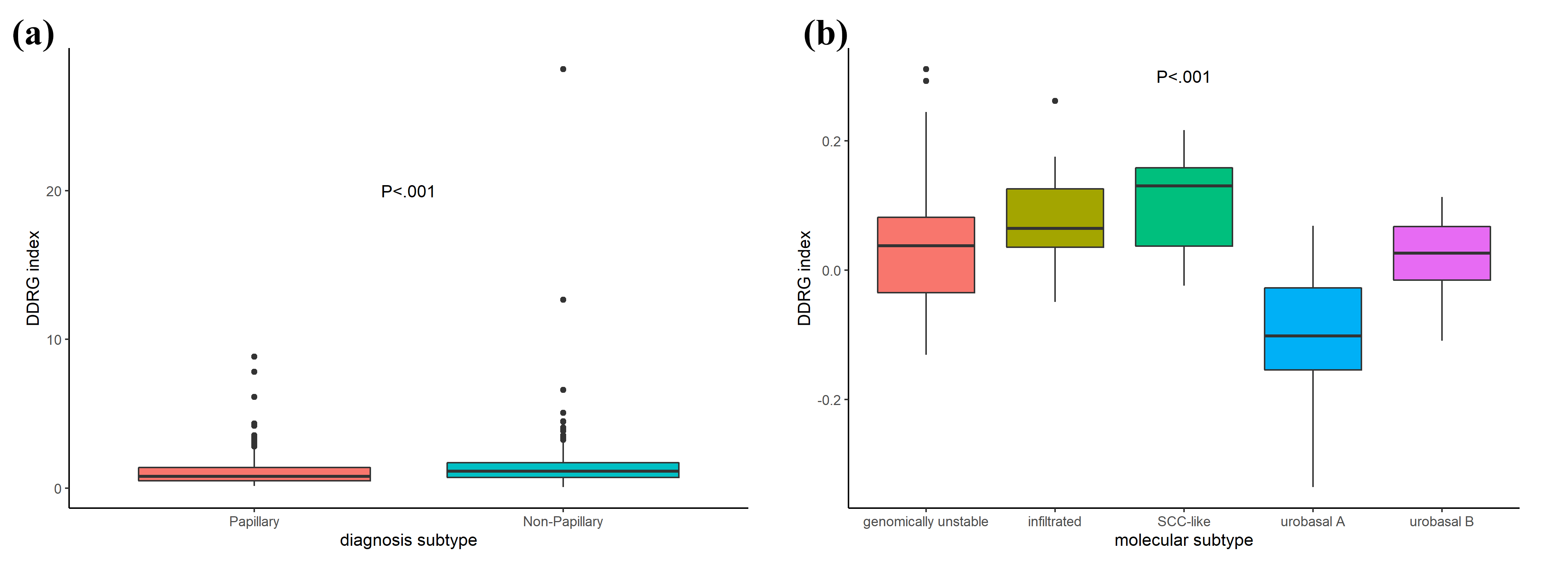

Supplement: Supplementary file 9 [file Image8.TIF]

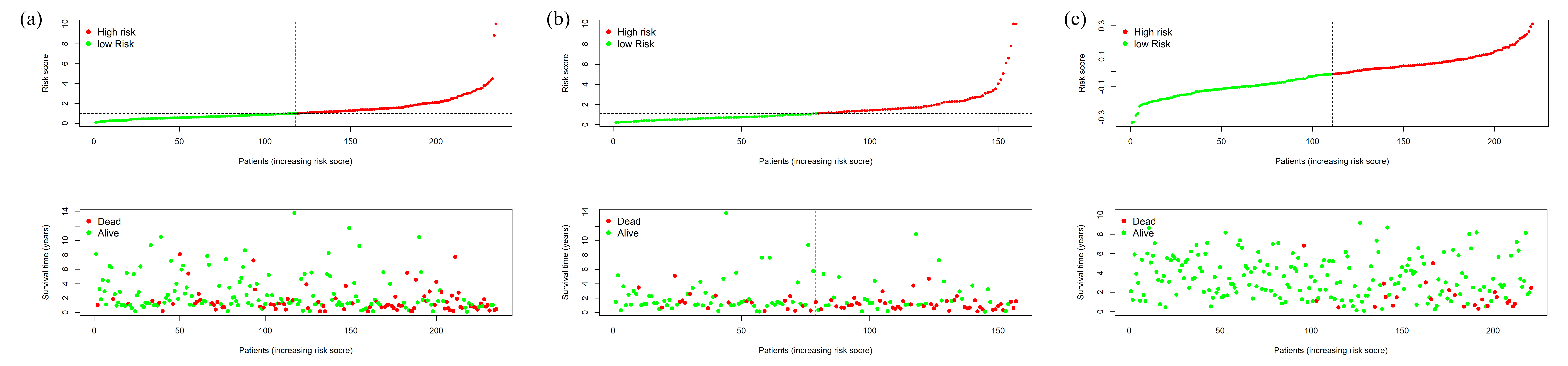

Supplement: Supplementary file 10 [file Image5.TIF]
