# Supplementary material for: Prognostic Analysis of Differentially Expressed DNA Damage Repair Genes in Bladder Cancer
Source: Pathol Oncol Res. 2022 May 24;28:1610267. doi: 10.3389/pore.2022.1610267 (PMC9172279; doi:10.3389/pore.2022.1610267)
Supplement: Supplementary file 7 [file Table1.doc]

**Table. S1. The functions of 22 OS-related DNA damage repair genes (DDRGs**) **genes presenting in the discussion section**

| Gene names | Functions | References |
| --- | --- | --- |
| ATXN1 | It is signalled to DNA damage by Ataxia Telangiectasia Mutated kinase | Suart CE, et al. 2021 |
| CAD | A major actomyosin binding protein that is critically implicated in the regulation of the microfilament network, and acts as an important modulator of various cell function | Chang KP, et al. 2021 |
| CDCA2 | Promote proliferation and migration of melanoma | [W-H Jin](https://pubmed.ncbi.nlm.nih.gov/?sort=date&term=Jin+WH&cauthor_id=32633378), et al. 2020 |
| CDK5R1 | A proline-directed serine/threonine kinase that phosphorylates a variety of substrate | Lee JH, et al. 2007 |
| FOXM1 | A transcriptional factor which plays an important role in oncogenesis | [Nathakan Klinhom-On](https://pubmed.ncbi.nlm.nih.gov/?sort=date&term=Klinhom-On+N&cauthor_id=33997388), et al. 2021 |
| HDAC10 | The homeostasis of lysosomes | Ridinger J, et al. 2018 |
| HDAC4 | An epigenetic reader and controller of enhancers and super-enhancers that supervise the senescence program | [Eros Di, et al. 2021](https://pubmed.ncbi.nlm.nih.gov/?sort=date&term=Di+Giorgio+E&cauthor_id=33966634) |
| ISG15 | May be the link between CLEC16A and downstream autoimmune, inflammatory processes. | [Heather S Hain](https://pubmed.ncbi.nlm.nih.gov/?sort=date&term=Hain+HS&cauthor_id=33927318), et al. 2021 |
| JDP2 | A member of the basic leucine zipper (bZIP) family of transcription factors | Avraham S, et al. 2019 |
| LATS2 | Large tumor suppressor kinase 2, phosphorylates and inactivates YAP/TAZ | Gu Y, et al. 2019 |
| LDLR | Inhibit carcinoma proliferation and metastasis by elevating intracellular cholesterol synthesis through MEK/ERK signaling pathway | Chen Z, et al. 2021 |
| MT1A | Been associated with the ability of free radical scavenging and detoxification of heavy metals leading to cancer development. | [R-R Rosa](https://pubmed.ncbi.nlm.nih.gov/?sort=date&term=Rosa+RR&cauthor_id=33340085), et al. 2021 |
| NEIL3 | Activate cell cycle progression | [Cui Zhao](https://pubmed.ncbi.nlm.nih.gov/?sort=date&term=Zhao+C&cauthor_id=33879165), et al. 2021 |
| ORC1 | Play a key role in the progression of bladder cancer via the Wnt/β-catenin pathway | Chen Z, et al. 2018 |
| PBK | A serine/threonine kinase, is a downstream target of RORγ that exerts the cellular effects | [Xiong Zhang](https://pubmed.ncbi.nlm.nih.gov/?sort=date&term=Zhang+X&cauthor_id=33916325), et al. 2018 |
| PDGFRA | An important gene in the IMATINIB pathway | [AE G](https://pubmed.ncbi.nlm.nih.gov/?sort=date&term=Ebrahimpour+Gorji+A&cauthor_id=33953878), et al. 2018 |
| POLA2 | DNA double strand breaks (DSB) repair and resistance to genotoxic stress | Dang TT, et al. 2020 |
| RRM2 | Malignant biological behaviors and activation of cGAS/STING | [Xueping Jiang](https://pubmed.ncbi.nlm.nih.gov/?sort=date&term=Jiang+X&cauthor_id=33858512),et al. 2021 |
| SREBF1 | A gene coding for a transcription factor related to cholesterol and fatty acid synthesis | [Inés Irurzun](https://pubmed.ncbi.nlm.nih.gov/?sort=date&term=Irurzun+I&cauthor_id=33742461),et al. 2021 |
| STAT1 | A key gene in a gene regulatory network related to immune phenotypes in bladder cance | Weng H, et al. 2021 |
| TACC1 | Perform a complex in the nucleus that binds specific retinoic acid response elements | JJ W,et al. 2017 |
| THBS1 | A membrane signal receptor and activator of the cAMP signaling pathway | [Fu-Mei Duan](https://pubmed.ncbi.nlm.nih.gov/?sort=date&term=Duan+FM&cauthor_id=33997182) , et al. 2021 |
