# Supplementary material for: Prognostic Analysis of Differentially Expressed DNA Damage Repair Genes in Bladder Cancer
Source: Pathol Oncol Res. 2022 May 24;28:1610267. doi: 10.3389/pore.2022.1610267 (PMC9172279; doi:10.3389/pore.2022.1610267)
Supplement: Supplementary file 8 [file Table2.docx]

**Table. S2. The functions of 7 core TFs genes presenting in the discussion section**

| Gene names | Functions | References |
| --- | --- | --- |
| NCAPG | Cell proliferation (for example, spindle, mitotic nuclear division and microtubule binding | Pan S, et al. 2019 |
| DNMT1 | Participates in DNA modification and activates/silences genes accompany with demethylation/methylation | Song C, et al. 2018 |
| LMNB1 | Plays an significant effect in regulation of gene expression, neural development and nuclear envelope stability | Cristofoli F, et al. 2020 |
| BRCA1 | a tumor suppressor responsible for DNA synthesis in normal cells | Romagnolo AP, et al. 2015 |
| E2H2 | Be implicated in carcinogenesis, and its downregulation has a therapeutic effect on B-cell lymphomas | Agrawal R, et al. 2020 |
| CENPA | An epigenetic marke | Saha AK, et al. 2020 |
| E2F7 | A critical player that transcriptionally regulates cell-cycle genes | P. Khongkow, et al. 2016 |
